# Memory complaints at primary care in a middle-income country: clinical and neuropsychological characterization

**DOI:** 10.1590/1980-57642021dn15-010009

**Published:** 2021

**Authors:** Marcos Leandro Pereira, Thiago Henrique Ferreira de Vasconcelos, Amanda Aparecida Rocha de Oliveira, Sarah Bárbara Campagnolo, Sarah de Oliveira Figueiredo, Ana Flávia Bereta Coelho Guimarães, Maira Tonidandel Barbosa, Luís Felipe José Ravic de Miranda, Paulo Caramelli, Leonardo Cruz de Souza

**Affiliations:** 1 Programa de Pós-Graduação em Neurociências, Universidade Federal de Minas Gerais – Patos de Minas, MG, Brazil.; 2 Curso de Medicina, Centro Universitário de Patos de Minas – Patos de Minas, MG, Brazil.; 3 Curso de Psicologia, Centro Universitário de Patos de Minas – Patos de Minas, MG, Brazil.; 4 Departamento de Clínica Médica, Faculdade de Medicina, Universidade Federal de Minas Gerais – Belo Horizonte, MG, Brazil.

**Keywords:** memory, primary health care, cognitive dysfunction, dementia, memória, atenção primária à saúde, comprometimento cognitivo, demência

## Abstract

There are different causes of memory complaints in the elderly, such as subjective cognitive decline (SCD), mild cognitive impairment (MCI) or dementia.

**Objective:**

1) To characterize individuals with memory complaints in a mid-sized city in Brazil, through clinical, cognitive and functional assessment; 2) to compare SCD individuals with MCI and dementia patients in terms of clinical and cognitive variables.

**Methods:**

We consecutively included individuals aged ≥50 years, with memory complaints (spontaneous or inquired). Subjects who scored ≥25 on the Memory Complaint Questionnaire or who had spontaneous memory complaints were selected. Participants underwent a semi-structured interview, the Mini-Mental State Examination, Figure Memory Test for visual episodic memory, Clock Drawing Test, Category Fluency (Animals), Neuropsychiatric Inventory, and functional assessment. Individuals were classified as SCD, MCI or dementia. We did not include individuals with previous diagnosis of dementia.

**Results:**

The final sample consisted of 91 subjects (73.6% women; mean age 67.6±9.8 years): 14.3% had spontaneous complaints and 85.7% had inquired complaints. The most common comorbidities were hypertension (69.2%), diabetes (36.3%), and dyslipidemia (24.2%). Low levels of vitamin B12 and hypothyroidism were found in 26.4 and 16.5%, respectively. Regarding cognitive diagnosis, 16.5% of the sample were classified as SCD, 49.4% as MCI and 34.1% as dementia. MCI and dementia were identified in five (38.5%) and seven (53.4%) patients with spontaneous complaint, respectively.

**Conclusions:**

MCI and dementia are frequently underdiagnosed. Potential reversible causes of cognitive decline are common. The diagnosis of dementia is highly frequent among individuals with spontaneous memory complaints.

## RESUMO.

Há diferentes causas de queixas de memória nos idosos, como declínio cognitivo subjetivo (DCS), comprometimento cognitivo leve (CCL) ou demências.

**Objetivo:**

1) Caracterizar indivíduos com queixa de memória em uma cidade de médio porte do Brasil, por meio de avaliação clínica, cognitiva e funcional; 2) comparar indivíduos com DCS, com CCL e pacientes com demência em termos de variáveis clínicas e cognitivas.

**Métodos:**

Incluiu-se, de modo consecutivo, indivíduos com idade ≥50 anos, com queixas de memória (espontânea ou inquirida). Foram selecionados participantes que pontuaram ≥25 no Questionário de Queixa de Memória ou que apresentaram queixa de memória espontânea. Todos foram submetidos à entrevista semiestruturada, Miniexame do Estado Mental, Teste de Figuras (teste de memória episódica visual), Teste do Desenho do Relógio, Fluência Semântica (Animais), Inventário Neuropsiquiátrico e avaliação funcional. Os indivíduos foram classificados em declínio cognitivo subjetivo (DCS), CCL e demência.

**Resultados:**

A amostra final foi composta por 91 indivíduos (73,6% mulheres; média de idade 67,6±9,8 anos); 14,3% apresentaram queixa espontânea e 85,7%, queixa inquirida. As comorbidades mais comuns foram hipertensão (69,2%), diabetes (36,3%) e dislipidemia (24,2%). Baixos níveis de vitamina B12 e hipotireoidismo foram encontrados em 26,4 e 16,5%, respectivamente. Quanto ao diagnóstico cognitivo, 16,5% foram classificados como DCS, 49,4% como CCL e 34,1% como demência. CCL e demência foram respectivamente identificados em cinco (38,5%) e sete (53,4%) pacientes com queixa espontânea de memória.

**Conclusões:**

CCL e demência são frequentemente subdiagnosticados. Causas potencialmente reversíveis de declínio cognitivo foram frequentes na amostra. O diagnóstico de demência foi muito frequente entre indivíduos com queixas espontâneas de memória.

## INTRODUCTION

Cognitive complaints are frequent among older adults.^1^ Some cognitive functions tend to decrease with age, such as attention and executive functions.^2^ During normal aging, episodic memory may also be affected by encoding or recall deficits, which depend on executive functions.^3^ Indeed, declining processing speed, reduced processing resources, and decreased cognitive control may account for age-related memory complaints.^2^

On the other hand, memory loss is a frequent symptom in different neuropsychiatric disorders, including dementias and psychiatric disorders,^4^ and is also found in systemic conditions (e.g., hypothyroidism and vitamin B12 deficiency).^5^

Previous studies investigated the prevalence of memory complaints in different populations. The frequency of memory complaints is variable across studies, ranging from 8 to 50%.^6^ ^–^ ^8^ Older age, female sex, depressive and anxious symptoms and low educational level are generally associated with a higher prevalence of memory complaints.^3^ ^,^ ^9^ ^,^ ^10^ Moreover, memory complaints can predict dementia, particularly in patients with mild cognitive impairment (MCI).^11^ ^,^ ^12^ Despite their clinical relevance, memory complaints are not always reported to the general practitioner.^12^

Complaints of memory loss may be associated with subjective cognitive decline (SCD), which is currently defined by two hallmark features: 1) self-experience of continuous deterioration in cognitive status, in comparison with the individual's preceding level and; 2) normal performance on standardized neuropsychological tests, considering education, age and gender.^13^ SCD is not merely an age-related phenomenon but is recognized as an important risk factor for MCI and Alzheimer's disease (AD).^14^

Considering that cognitive disorders may be due to reversible causes, it is crucial that health professionals perform proper cognitive screening at primary care. Moreover, as SCD and MCI are risk factors for AD, the detection of these conditions at primary care can contribute to early interventions, thus changing the outcome of clinical conditions related to memory loss.

Despite this clinical relevance, there are few studies of SCD in primary health care,^15^ and most of them were conducted in populations from high-income countries, with high educational level.^16^ There is scarce data about cognitive impairment and memory complaints in low- and middle-income countries,^10^ ^,^ ^16^ ^–^ ^19^ especially in primary health care. As a matter of fact, the number of patients with dementia is globally increasing, especially in low and middle-income regions, such as Latin America,^20^ making it crucial to provide data about SCD and memory complaints in these populations.

The objective of this study was to characterize individuals with memory complaints in a mid-sized city in Minas Gerais State, Brazil, through a comprehensive clinical, cognitive and functional assessment. We also aimed to compare SCD individuals with MCI and dementia patients in terms of clinical and cognitive variables.

## METHODS

This was an observational, cross-sectional study. Data collection was carried out from March to September 2016, at the Lagoa Grande Basic Health Unit in Patos de Minas. Patos de Minas is located in Minas Gerais (Southeast region, Brazil), with a population of 152,488 inhabitants. The Human Development Index (HDI, 2020) of the municipality of Patos de Minas is 0.765, which is slightly higher than that of Brazil (0.755, world rank 75th). Patos de Minas’ gross domestic product (GDP-2019) per capita is about US$ 5,182, which is lower than Brazil's index in 2019 (US$ 6,155).

**Figure 1:**
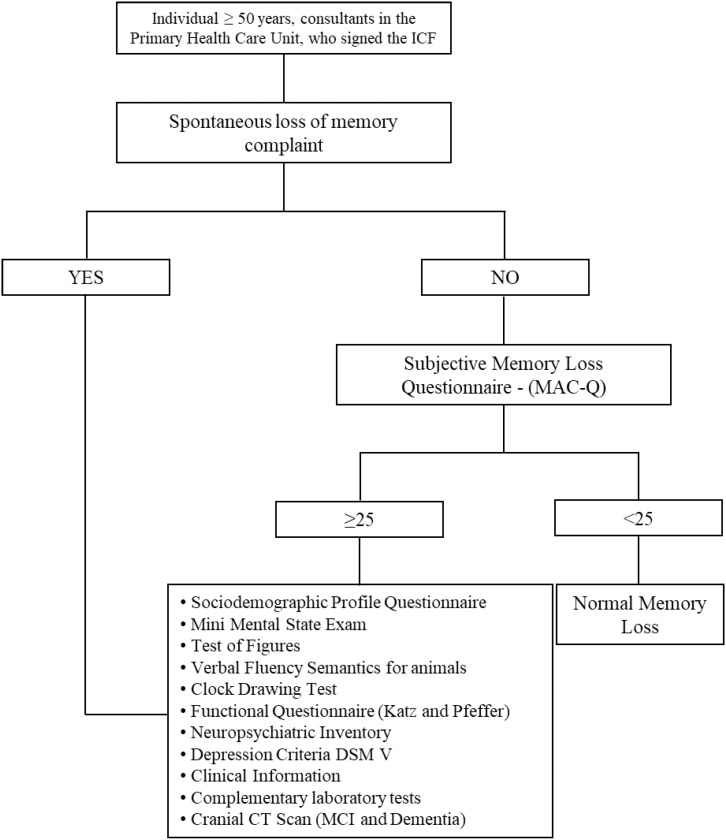
Study design.

This study was proposed for individuals over 50 years of age who were consecutively seen at a general practice visit. Importantly, we did not include participants with previously established diagnosis of dementia. Figure 1 shows the flowchart of the study.

Subjects who spontaneously presented with memory complaints (as the main reason for the consultation, hereafter referred to as “spontaneous memory complaints”) were submitted to neuropsychological and laboratory investigation. In turn, subjects who had no spontaneous complaint of memory deficits were asked about the functioning of memory with an open question (“How is your memory?”). Those who answered with memory complaints completed the Memory Complaint Questionnaire (MAC-Q),^16^ and those who scored 25 points or more on the MAC-Q were referred for neuropsychological and laboratory investigation. Individuals with no spontaneous memory complaints but who scored 25 or more on the MAC-Q are hereafter referred to as subjects with “inquired memory complaints”.

Participants underwent a semi-structured questionnaire, describing sociodemographic and medical variables (comorbidities, use of medications, alcohol use, smoking, physical activity). Next, the Brief Cognitive Battery^21^ was applied, which includes the Mini-Mental State Examination (MMSE)^22^, the Figure Memory Test (FMT, a Visual Episodic Memory Test),^18^ the Clock Drawing Test^23^ and Verbal Fluency (Animal Category).^24^ Participants were also submitted to the Neuropsychiatric Inventory (NPI)^25^ and Functional Activity Questionnaire (FAQ)^26^ (Figure 1). All participants were submitted to laboratory tests to investigate cognitive decline, according to current recommendations.^27^

Based on clinical and neuropsychological data, subjects were classified into three clinical categories: 1) SCD (MAC-Q ≥25, with no change in neuropsychological tests and no functional decline, FAQ ≤5); 2) MCI, with abnormal score in one of the cognitive tests (abnormal MMSE, FMT-Recall 5’,<7, abnormal Verbal Fluency or Clock Drawing Test, <4), and preserved functional capacity, FAQ ≤5; and 3) dementia, with abnormal cognitive scores and functional impairment — FAQ >5). The normal cut-off scores for the MMSE^22^ ^,^ ^24^ and for Verbal Fluency were extracted from normative data for the Brazilian population, considering the educational level as follows: MMSE:^22^ 20 — illiterate; 25 — 1 to 4 years of schooling; 26 — 5 to 8 years of schooling; 28 — 9 to 11 years of schooling and 29 — more than 11 years of schooling; Verbal Fluency Test – Animals:^24^ 11 for illiterate, 13 for 1 to 4 years of study and 14 for more than 5 years of study.

Patients of the MCI and dementia groups were referred to brain computerized tomography (CT), without contrast, to investigate structural brain lesions. We did not include patients with structural brain lesions (e.g., brain tumor, subdural hematoma).

The study was approved by the Local Ethics Committee of the University Center of Patos de Minas (No. 1.733.241). All participants or their relatives, when necessary, signed an informed consent form after clarification.

### Statistical analyses

All statistical analyses were performed using *Statistical Package for the Social Sciences* (SPSS) 22.0 (SPSS Inc., Chicago, IL). Qualitative variables were described according to frequencies and percentages. The normality of the quantitative variables was verified with the Shapiro-Wilk test, after visual inspection of the histograms. The chi-square test was used for comparing frequencies between groups. Comparisons between continuous variables with normal distribution were analyzed by ANOVA, followed by the Tukey test. For non-normal distribution continuous variables, the Mann-Whitney test (for two independent groups) or the Kruskal-Wallis test (for comparison of multiple groups, followed by the Dunn post-test, when appropriate) was used. We adopted Bonferroni's correction for multiple comparisons and the level of significance (α) was set at 0.004.

## RESULTS

### Descriptive analysis (total population)

**Figure 2:**
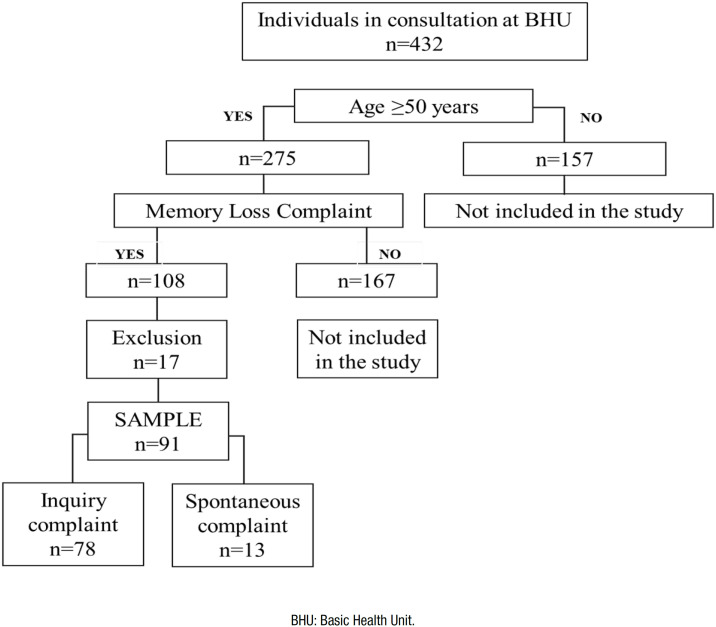
Sample flowchart

During the study period, 432 individuals were referred for medical consultation at the Lagoa Grande Basic Health Unit, with 275 of them considered “initial sample” with age equal to or greater than 50 years. Among these, 167 subjects did not present with memory complaints (either spontaneous or inquired), while 108 (39% of the initial sample) had either spontaneous or inquired memory complaints. Seventeen subjects were excluded for the following reasons: (refused to undergo cognitive tests) (n=8), brain tumor (n=1), changed area covered by the health unit (n=3) and score <25 on MAC-Q (n=5). The final sample of the study consisted of 91 participants (Figure 2), being 13 (14.3%) with spontaneous memory complaints and 78 (85.7%) with inquired memory complaints.

Table 1 presents the demographic data of the sample. Women (74.7%), with mean age of 67.6 years (±9.8), composed most of the study population. Most individuals had low educational level (4–8 years of schooling — n=47, 51.7%).

**Table 1:** Demographic data (n, %) for the study population, according to clinical group.

		SCD (n=15)	MCI (n=45)	Dementia (n=31)	Total (n=91)
**Sex** ¥	Female	8 (53.3%)	35 (77.8%)	25 (80.6%)	68 (74.7%)
	Male	7 (46.7%)	10 (22.2%)	6 (19.4%)	23 (25.3%)
**Age (mean±standard deviation)** §		64.9±9^a^	66.4±8.9	70.6±10.8	67.6±9.7
**Schooling (years)** ¥	Illiterates	1 (6.7%)	2 (4.4%)	4 (12.9%)	7 (7.7%)
	1 to 3	5 (33.2%)	11 (24.5%)	8 (25.8%)	24 (26.4%)
	4 to 8	7 (46.7%)	24 (53.3%)	16 (51.6%)	47 (51.6%)
	9 to 11	1 (6.7%)	3 (6.7%)	1 (3.2%)	5 (5.5%)
	>11	1 (6.7%)	5 (11.1%)	2 (6.5%)	8 (8.8%)
**Family income (in Brazilian minimum wage)** ¥	1 to 2	11 (3.3%)	27 (60.0%)	16 (51.6%)	54 (59.3%)
	3 to 5	4 (26.7%)	16 (35.6%)	14 (45.2%)	34 (37.4%)
	6 to 10	0 (0.0%)	2 (4.4%)	1 (3.2%)	3 (3.3%)
	>10	0 (0.0%)	0 (0.0%)	0 (0.0%)	0 (0.0%)
**Civil status** ¥	Unmarried	3 (20.0%)	2 (4.4%)	3 (9.7%)	8 (8.8%)
	Married	10 (66.7%)	24 (53.3%)	17 (54.8%)	51 (56.0%)
	Widow	2 (13.3%)	12 (26.7%)	8 (25.8%)	22 (24.2%)
	Divorced	0 (0.0%)	7 (15.6%)	3 (9.7%)	10 (11.0%)

MCI: mild cognitive impairment; SCD: subjective cognitive decline. The p-values refer to the significance level of the comparison between the 3 groups.

¥ The frequency of qualitative variables (sex, schooling, family income and civil status) was compared between groups with the chi-square test.

§ The quantitative variable (age) between the groups was compared using the Kruskal-Wallis test.

a p<0.05 (subjective complaint vs dementia).

Table 2 shows comorbidities and medications in use for the study population. Systemic arterial hypertension was present in 69%; diabetes, dyslipidemia and hypothyroidism were found in 36.3, 25.3 and 22%, respectively. Regarding medications, 33% of participants used antidepressants or anxiolytics (mainly selective serotonin reuptake inhibitors) and 23% used a proton pump inhibitor. Laboratory analyses found that 26.4% had low levels of vitamin B12, and 16.5% had thyroid-stimulating hormone (TSH) levels above normal. None of the individuals tested positive for human immunodeficiency virus (HIV) or syphilis. Most patients (MCI and dementia) did not show abnormalities on CT scan. Leukoaraiosis was found in 7.6% of MCI or patients with dementia, being more frequent in the dementia group.

**Table 2:** Clinical data (medications and comorbidities) for the study population, according to clinical group (n, %).

		SCD (n=15)	MCI (n=45)	Dementia (n=31)
**Medications**				
	Proton pump inhibitor	2 (13.3%)	9 (20%)	10 (32.2%)
	Antidepressant	4 (26.7%)	12 (26.7%)	14 (45.2%)
	Typical antipsychotic	0 (0%)	1 (2.2%)	1 (3.2%)
	Atypical antipsychotic	0 (0%)	0 (0%)	1 (3.2%)
	Benzodiazepine p<0.015	3 (20.0%)	6 (13.3%)^a^	13 (41.9%)
**Comorbidities**				
	Alchool use	2 (13.3%)	8 (17.7%)	2 (6.4%)
	Smoking	2 (13.3%)	5 (11.1%)	3 (9.7%)
	Hypertension	11 (73.3%)	28 (62.2%)	24 (77.4%)
	Diabetes	5 (33.3%)	17 (37.8%)	11 (35.5%)
	Dyslipidemia	6 (40.0%)	12 (26.7%)	5 (16.1%)
	Hypothyroidism	4 (26.7%)	9 (20.0%)	7 (22.6%)

SCD: subjective cognitive decline; MCI: mild cognitive impairment;

a p<0.05 (dementia vs MCI).

### Comparison of groups: spontaneous vs inquired memory complaints

Among subjects with memory complaints (n=91), 13 (14.3%) had spontaneous memory complaints and 78 (85.7%) had inquired memory complaints. Individuals from these groups did not differ in age, sex distribution, schooling and family income (Supplementary Table). The two groups did not differ in the frequency of clinical diseases (hypertension, diabetes, dyslipidemia, and hypothyroidism).

Regarding the cognitive diagnoses, SCD, MCI and dementia were identified in one (7.7%), five (38.5%) and seven (53.4%) subjects with spontaneous memory complaints, respectively. In the group with inquired memory complaints, 12 (15.4%), 42 (53.4%) and 24 (30.8%) had SCD, MCI and dementia, respectively.

Table 3 presents the cognitive data of all participants. Compared to subjects with inquired memory complaints, those with spontaneous memory complaints had lower scores on the MMSE, but without statistical significance (p=0.015). These groups did not differ in 5’Recall (FMT), Animal Fluency and Clock Drawing Test.

**Table 3:** Neuropsychological data (mean±standard deviation) for clinical groups according to complaint type.

	Spontaneous complaint (n=13, 14.3%)	Inquired complaint (n=78, 85.7%)
MAC-Q p<0.05	31.1±3.7	29.3±3.0^b^
MMSE p<0.01	19.8±3.8	22.8±4.5^a^
Figure Memory Test (Recall 5’) p<0.05	5.2±3.2	7.0±2.2^b^
Verbal Fluency (Animals)	8.3±2.8	9.8±3.5
Clock Drawing Test	3.5±1.7	3.2±1.9
FAQ	3.0±2.7	2.4±2.6

FAQ: Functional Activity Questionnaire; MAC-Q: Memory Complaint Questionnaire; MCI: mild cognitive impairment; MMSE: Mini-Mental State Examination; SCD: subjective cognitive decline. The comparison between groups was performed using the Mann-Whitney test;

a p<0.01 (spontaneous vs surveyed);

b p<0.05 (spontaneous vs respondent).

### Comparison of groups: subjective cognitive decline, mild cognitive impairment and dementia

According to clinical criteria, participants (n=91) were clinically categorized as follows: 15 (16.5%) with SCD, 45 (49.4%) with MCI and 31 (34.1%) with dementia.

These three groups did not differ in age, sex distribution, schooling and family income. There was no statistically significant difference in the frequency of comorbidities (hypertension, diabetes, dyslipidemia, and hypothyroidism) between groups. The frequency of use of antidepressants, antipsychotics and proton pump inhibitors did not differ across groups, but there was a trend (p<0.015) for a higher frequency of benzodiazepine use in the dementia group, when compared to the MCI group. Participants with regular physical activity were 8.3, 31.2 and 16.1% in the SCD, MCI and dementia groups, respectively.

Table 4 presents cognitive tests and scales for the clinical groups (SCD, MCI and dementia). There was a statistically significant difference in MMSE between groups, with higher scores in the SCD group, and lower in the dementia group, with MCI showing intermediate scores (SCD<MCI<dementia). Similarly, individuals with dementia performed worse than MCI (p<0.002) and SCI (p<0.001) individuals in the 5’Recall (FMT). Compared to SCD and MCI, patients with dementia also underperformed on Animal Fluency and on the Clock Drawing Test. Individuals with SCD and MCI had similar performances on all cognitive tests, except for MMSE (p<0.001), with lower scores in the MCI group.

**Table 4:** Neuropsychological data (mean±standard deviation) for the study population, according to clinical group.

	SCD (n=15)	MCI (n=45)	Dementia (n=31)
MAC-Q	29.3±2.9	29.0±2.8	30.4±3.7
MMSE p<0.001	26.7±1.8^a^,^b^	23.4±3.4^a^	18.7±4.3
Figure Memory Test (Recall 5’) p<0.001	8.1±1.1^a^	7.3±2.0^a^	5.3±2.7
Verbal Fluency (Animals) p<0.001	12.4±1.8^a^	9.9±3.5	7.7±2.9
Clock Drawing Test	3.9±1.3	3.6±1.6	2.3±2.0
FAQ	1.0±1.3	1.0±1.0	12.5±7.9

FAQ: Functional Activity Questionnaire; MAC-Q: Memory Complaint Questionnaire; MCI: mild cognitive impairment; MMSE: Mini-Mental State Examination; SCD: subjective cognitive decline;

a significant difference vs dementia group (p<0.004; Mann-Whitney test);

b significant difference vs MCI group (p<0.004; Mann-Whitney test).

The scores on the NPI across the groups are shown in Table 5. The groups did not differ in the following scores: disinhibition, dysphoria, anxiety, irritability, aberrant motor behavior, euphoria and night-time behavioral disturbances. Dementia group had higher scores than SCD on the NPI total score (p<0.002). Compared to MCI group, patients with dementia scored higher on apathy (p<0.001), agitation (p<0.003), appetite disorders (p<0.004) and on the NPI total score (p<0.001). The groups SCD and MCI did not differ in any of NPI scores.

**Table 5:** Scores (mean±standard deviation) on the Neuropsychiatric Inventory, according to clinical group.

Neuropsychiatric Inventory	SCD (n=15)	MCI (n=45)	Dementia (n=31)
Hallucinations	0.0±0.0	0.0±0.0	0.4±1.7
Delusions	0.0±0.0	0.0±0.0	0.4±1.5
Apathy	0.2±0.6	1.2±2.2^a^	4.3±4.7
Dysphoria	2.5±3.2	2.5±3.55	4.9±3.9
Agitation/aggression	0.1±0.3	0.6±2.1^a^	3.2±4.3
Anxiety	3.4±4.7	2.8±3.6	5.4±4.7
Disinhibition	0.1±0.3	0.19±0.55	0.4±1.2
Irritability/lability	1.4±2.7	1.7±3.4	3.3±4.6
Aberrant motor activity	0.0±0	0.03±2.15	0.6±2.0
Euphoria	0.0±0	0.03±2.15	0.3±1.5
Appetite and eating abnormalities	0.5±1.7	0.85±2.3^a^	3.7±4.8
Night-time behavioral disturbances	0.0±0	0.6±2.26	1.3±3.4
Total score	11.1±16.5^a^	10.2±13.2^a^	28.4±21.6

MCI: mild cognitive impairment; SCD: subjective cognitive decline;

a significant difference vs dementia group (p<0.004; Mann-Whitney test).

## DISCUSSION

This study aimed to characterize complaints of memory loss in adults in primary health care in a middle-income country (Brazil). We found that clinical diseases, such as systemic arterial hypertension and hypothyroidism, were frequently observed in patients with memory complaints. We also identified notable frequencies of underdiagnosis of dementia and of treatable causes of cognitive decline among individuals with memory complaints. Finally, as an original contribution, we provide clinical characterization of subjects with SCD at primary health care in a middle-income country.

In line with previous studies,^1^ ^,^ ^10^ ^,^ ^11^ ^,^ ^12^ ^,^ ^28^ most of our sample was composed of women. The reasons for this are unclear, but both medical and sociological issues may account for this^28^. Women look for medical care more frequently than men,^28^ and it is possible that the memory loss in men is underdiagnosed.

There is scarce data about cognitive impairment and memory complaints in low- and middle-income countries, especially in primary health care. Data from India and China found respectively 10.8 and 17% of patients with cognitive impairment in primary health care surveys.^17^ ^,^ ^29^ The prevalence of memory complaints and cognitive impairment is heterogeneous across studies, ranging from 8 to 50%.^6^ ^–^ ^8^ Methodological issues, such as the target population (primary care, secondary outpatient clinics or referral centers) and differences on cognitive screening tests may explain the variability of the results across studies. Interestingly, the frequency of memory complaints in our sample is similar to that observed in study conducted at a high-income country.^30^ Waldorff and colleagues^30^ reported that 24% of Danish patients seen at primary health care had memory complaints. However, that study selected patients at least 65 years-old, while our study included individuals over 50 years of age. It is possible that we would find higher frequencies of memory complaints if we had included only patients over 65.

A previous study with elderly Brazilians community-living setting found that memory complaints were associated with low schooling and depressive symptoms but did not correlate with cognitive performance.^10^ Another Brazilian study found that the score on the Memory Complaints Scale correlated with the MMSE and with measures of visual-spatial abilities and orientation.^18^

This study investigated both inquired and spontaneous memory complaints. We found that inquired memory complaints (n=78; 85.7%) were more frequent than spontaneous memory complaints (n=13; 14.3%), in line with Burmester et al.,^31^ who reported that the frequency of spontaneous memory complaints was lower than when actively investigated with a structured questionnaire, in a large survey with 421 individuals. Again, these results raise the problem of underdiagnosis of cognitive decline in primary care.

Interestingly, subjects with spontaneous memory complaints had similar performance than those with inquired memory complaints on the cognitive tests. However, the frequency of dementia tended to be higher among participants with spontaneous memory complaints (53.4%) than in those with inquired memory complaints (30.8%). These results suggest that individuals with spontaneous memory complaints should be carefully screened for cognitive decline and dementia.

Previous diagnosis of dementia was an exclusion criterion, but 31 patients (34.1%) with memory complaints (seven with spontaneous memory complaints and 24 with inquired complaints) fulfilled criteria for dementia. All dementia patients had mild to moderate functional impairment, and none had severe dementia. These findings are in agreement with a recent meta-analysis showing that the underdiagnosis of dementia is high, with middle-income countries showing higher rates (above 90%) than in high-income countries (around 60%).^32^ These results reinforce the urgent need for dementia screening at primary care.

We identified 15 out 91 (16.5%) individuals with SCD, i.e., patients who had memory complaints but with no objective deficit on cognitive assessment.^33^ To the best of our knowledge, this is one of the first reports of frequency of SCD in a middle-income country.^18^ Considering that SCD is associated with increased risk for developing MCI and AD,^14^ a clinical follow-up of these individuals is warranted.

In agreement with previous studies, non-communicable chronic diseases such as systemic arterial hypertension, diabetes mellitus and dyslipidemia were frequently observed in our sample.^34^ ^–^ ^36^

Interestingly, there was no difference in frequencies of these comorbidities across groups of SCD, MCI and dementia. The interactions between these conditions and cognitive performance are a matter of debate. A Brazilian study did not find a significant difference in verbal fluency between healthy controls and patients with hypertension and/or diabetes,^37^ but other studies found an association between poor cognitive performance and cardiometabolic diseases such as hypertension, diabetes and obesity.^37^ ^,^ ^38^ Importantly, schooling may modulate the impact of these diseases in cognitive performance, as education is associated with adherence to preventive measures.^39^

Of note, we found substantial percentages of patients with potential reversible causes of cognitive decline. For instance, 26.4% of participants with memory complaints had low levels of vitamin B12, and 16.5% had abnormal TSH levels. These data reinforce the need for comprehensive screening for non-degenerative causes of cognitive deficit at primary care settings.

Patients with dementia had higher scores on the NPI than SCD and MCI patients. The use of benzodiazepines tended to be more frequent among patients with dementia thus suggesting that these medications were prescribed for treating neuropsychiatric symptoms associated with dementia. There is evidence that long-term use of benzodiazepines is associated with an increased risk of dementia,^40^ ^–^ ^42^ although this association may be considered causal.^42^ ^,^ ^43^ Considering the cross-sectional design of our study, we cannot establish a causal relation between benzodiazepine use and dementia. On the other hand, these medications are associated with increased risk of falls^40^ and impairment in executive abilities^41^ in patients with dementia. Our data support the need for a detailed inventory of medications in use in patients with memory complaints, to avoid possible negative effects, especially in patients with established dementia.

This study has some limitations. Besides the small sample size, we did not include a control group, without memory complaints, which would be of value for comparative purposes. Considering that we employed a self-scale for screening of memory complaints, it is possible that subjects with anosognosia were not retained in the study. Moreover, the value of the MAC-Q as a screening tool is limited by the interference of the affective status,^44^ but the MAC-Q has been successfully used in the Brazilian population.^10^ The diagnosis was established on clinical grounds, and participants did not undergo formal neuropsychological examination and did not pass advanced investigation with brain magnetic resonance imaging and biological markers of AD. Therefore, we cannot establish the etiology for cognitive decline and dementia in our group. Finally, we adopted strict statistical correction for multiple comparisons, to avoid spurious results. It is possible that we would find more differences for clinical and cognitive variables between groups using a less strict level of significance (e.g., p<0.05).

Despite these limitations, this study provides relevant clinical information to general practitioners working at the primary health care level, as well as for public health programs. The early diagnosis of cognitive decline provides the best opportunities for care planning and medical assistance for patients and caregivers. In this context, physicians and the primary care team play a key role in the early recognition of cognitive impairment in their patients.^45^ We detected substantial percentages of non-diagnosed MCI and dementia among individuals with memory complaints, and we also found that potentially reversible causes of cognitive impairment, such as hypovitaminosis B12 and hypothyroidism, are frequent at primary care. We also identified that the diagnosis of dementia was very frequent among subjects with spontaneous memory complaints. Taken together, these results reinforce the central role of primary care assistance in the diagnosis and medical care of individuals with memory complaints. Finally, we suggest that physicians in primary health care be trained to diagnose MCI and dementia and to perform longitudinal monitoring of individuals with SCD as well.
